# Pleomorphic hyalinizing angiectatic tumour of the lower back – a rare case and review of the literature

**DOI:** 10.1093/jscr/rjad719

**Published:** 2024-01-10

**Authors:** Carlos Neblett, Kenneth Appiah, Tahjeme Lawrence, Malik Graham, Daynalee Wilson, Rory Thompson

**Affiliations:** Division of Plastic & Reconstructive Surgery, Department of Surgery, Kingston Public Hospital, Kingston JMAKN03, Jamaica; Division of Plastic & Reconstructive Surgery, Department of Surgery, Kingston Public Hospital, Kingston JMAKN03, Jamaica; Division of Plastic & Reconstructive Surgery, Department of Surgery, Kingston Public Hospital, Kingston JMAKN03, Jamaica; Division of Plastic & Reconstructive Surgery, Department of Surgery, Kingston Public Hospital, Kingston JMAKN03, Jamaica; Division of Plastic & Reconstructive Surgery, Department of Surgery, Kingston Public Hospital, Kingston JMAKN03, Jamaica; Department of Pathology, University Hospital of the West Indies, Kingston JMAAW15, Jamaica

**Keywords:** trunk, back, pleomorphic hyalinizing angiectatic tumour, soft tissue

## Abstract

Pleomorphic hyalinizing angiectatic tumour (PHAT) is a very rare low-grade indeterminate neoplasm of connective and other soft tissue, which is not known to metastasize though local recurrence has been documented. It most commonly presents in the lower extremities, but other anatomical locations have been described. This is the second known case of PHAT from the Caribbean region and adds to the limited reported cases of the condition in the literature.

## Introduction

Pleomorphic hyalinizing angiectatic tumour (PHAT) is a very rare entity of uncertain behaviour involving connective or other soft tissue as per the World Health Organization [1]. This tumour, often mistaken for other mesenchymal lesions such as malignant fibrous histiocytoma (MFH) and schwannoma, typically occurs within the subcutaneous tissues, especially in the lower limbs of adults of both sexes. There is no known metastatic capacity but variable local recurrence rates (33–50%) have been reported in the <100 cases documented in the literature [2, 3]. Though no consensus regarding management exists, wide local surgical excision with microscopically tumour-free resection margins seems to be a reasonable option [4]. We report the second known case of PHAT from the Caribbean region after reviewing the literature.

## Case presentation

A 41-year-woman of African descent presented to the plastic and reconstructive surgery outpatient department, with a lesion to the lower back for 2 years which starting growing over the last 10 months. The increasing size of the lesion was associated with fatigue, weight loss and localized haemorrhage.

**Figure 1:**
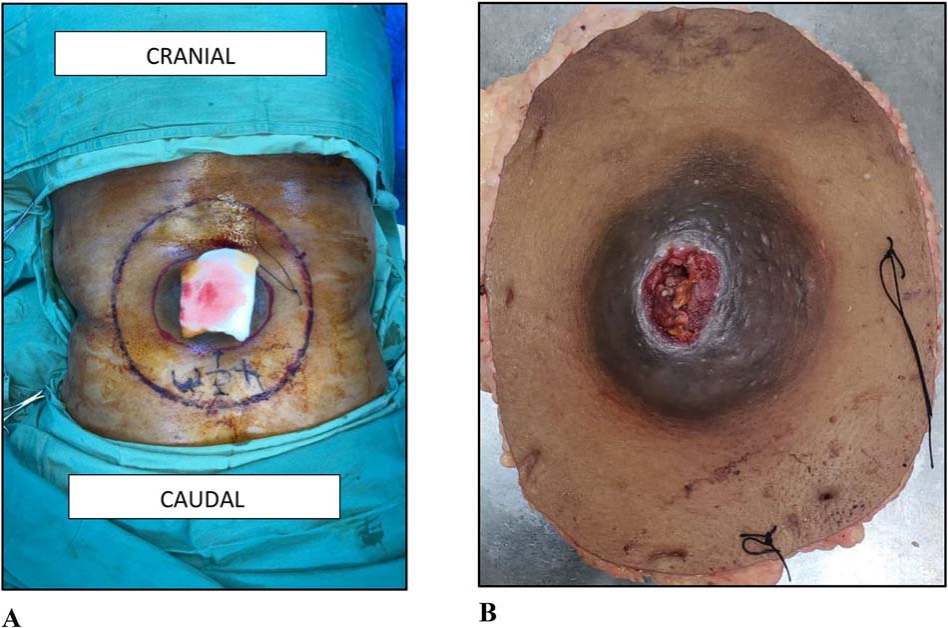
(A) Photograph of the lesion to the lower back. (B) Photograph of the wide local excision.

On examination, there was a 12.0 × 12.0 × 7.0 cm spherical, firm, smooth, mobile, non-tender, and ulcerating mass to the midline of the lower back 8.0 cm cranial to the natal cleft (Fig. 1). Clinically, the mass involved the subcutaneous plane and the overlying skin but not the underlying musculature. There was no associated groin lymphadenopathy.

Contrast-enhanced magnetic resonance imaging examination showed a well-defined 11.4 × 11.5 × 6.1 cm heterogenous midline subcutaneous lesion of the lower back neither involving the underlying muscle nor communicating with the spinal canal. Ultrasound imaging revealed significant intra-lesional vascular flow while no evidence of metastases was seen on staging computed tomographic scan.

Haematological test results revealed anaemia with haemoglobin count of 8.3 × 10^9^ /L (normal range is 12–16 × 10^9^/L).

**Figure 2:**
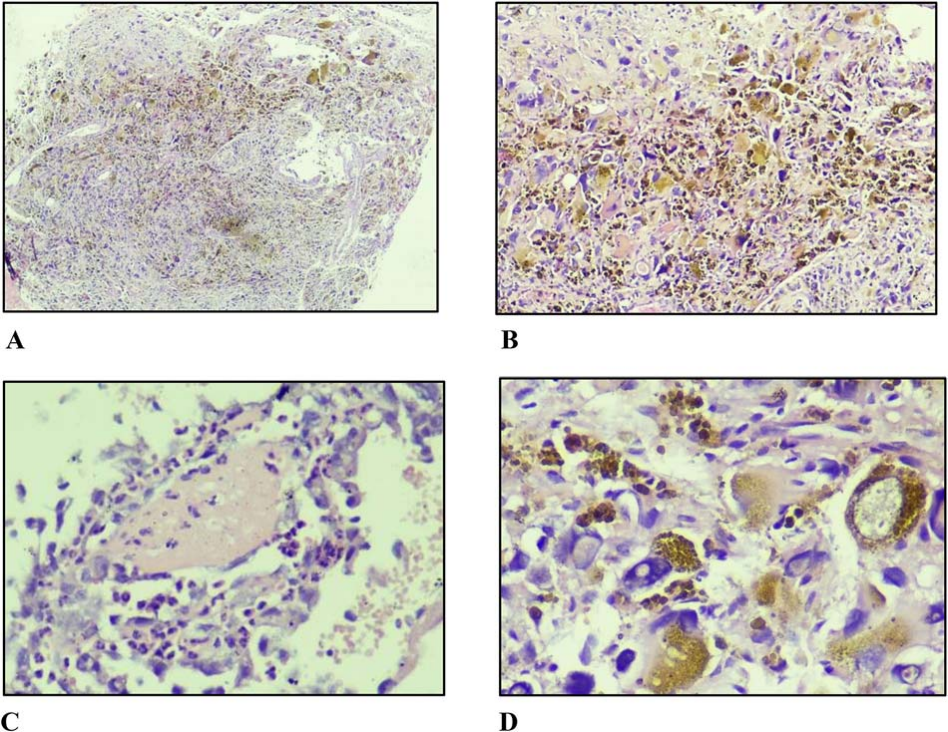
(A) Non-circumscribed lesion displaying sheet-like and fascicular growth of spindle and pleomorphic cells with haemosiderin deposition (×40). (B) Markedly pleomorphic cells in nondescript growth pattern with abundant haemosiderin deposition (×100). (C) Fibrin surrounded by inflammatory cells and atypical endothelial cells (×400). (D) Markedly pleomorphic cells with abundant eosinophilic cytoplasm and large, irregular, hyperchromatic nuclei with prominent intranuclear pseudo-inclusions (×400).

A core biopsy was performed. The histopathological findings were that of fibroadipose tissue infiltrated by a non-encapsulated pleomorphic tumour with microcystic stroma, glial-like containing ectatic thin-walled blood vessels of varying sizes. These blood vessels were lined by focally multi-layered endothelial cells with abundant amorphous eosinophilic fibrin-like material in the subendothelial stroma. There were cells of varying sizes ranging from small round to spindle-shaped with clusters of bizarre giant cells exhibiting markedly pleomorphic nuclei containing large intranuclear cytoplasmic inclusions evident in many places. Focally prominent intracytoplasmic haemosiderin deposition was seen in the cells closest to the blood vessels. There were rare mitoses seen (Fig 2). These features favoured PHAT but immunohistochemistry consultation was recommended for confirmation and to exclude the main differentials. The results of which were positive for CD34 while negative for SOX-10, desmin, and MDM2.

After a multidisciplinary team meeting, preoperative optimization followed by surgical management using 4.0 cm circumferential margins and resection of the corresponding deep fascia overlying the musculature was done. After histological assessment revealed uninvolved margins, negating adjuvant radiation therapy, the defect was resurfaced with a meshed 3:1 split thickness skin graft. The patient is undergoing long-term follow-up in the outpatient department.

## Discussion

PHAT of soft parts is an extremely uncommon benign neoplasm of uncertain differentiation, first described by Smith et al. in 1996. It typically occurs within the subcutaneous tissue, with intramuscular extension on occasions, affecting the lower extremities most frequently, though other anatomical sites have been described. Both sexes are affected with a female to male ratio of 4:3 and a wide age range (10–89 years of age) documented. Since its description there have been <100 cases reported in the literature [1, 5]. The largest of which was done by Folpe and colleagues in 2004 [6], where 41 cases were reviewed with variable local recurrence rates but no metastasis noted. Given this data and the additional literature, PHAT are less likely benign as originally described and are probably better categorized as indeterminate or borderline malignancies given their local aggressive nature and lack of metastatic capacity [2–4, 6, 7].

On gross histology, a PHAT is a well circumscribed, firm, unecapsulated mass with lobulated appearance and variegated cut surfaces which are tan to maroon in colour, with clusters of varying sized thin wall ectatic blood vessels containing intra-luminal fibrin deposition and thrombi. Microscopically, there is stroma; composed of sheets and fascicles of spindle shaped, pleomorphic and multinucleated giant cells containing intranuclear inclusions. The blood vessels with subendothelial fibrin deposition and perivascular hyalinization are scattered throughout this stroma and the cells closest to them contain fine haemosiderin deposition within their cytoplasm. Inflammatory mast cells, occasional myxoid areas and focal calcification are also present in the stroma. Despite the high pleomorphism, mitotic activity is usually minimal or completely absent [1, 4]. These characteristic features were present in this case.

The main differential diagnoses include MFH which differs due to; a high mitotic rate, no intranuclear inclusions and absence of CD34 expression and schwannoma which varies due to; encapsulation, verocay body formation, S100, SOX-10, and MDM2 expression [1]. Immunohistochemistry studies; positive expression of CD34, vascular endothelial growth factor and vimectin along with negative expression of S-100, SOX-10, desmin, MDM2, and smooth muscle actin are useful ancillary tests to confirm diagnosis [4]. In this report, the lesion was positive for CD34, while negative for SOX-10, MDM2, and desmin, thereby excluding the above two diagnoses.

There is no consensus regarding treatment. Surgical excision with tumour-free resection margins—which was done—given the high local recurrence rates, and long-term follow-up is recommended, which will be carried out in this case. When incomplete resection or involved margins occur adjuvant radiation therapy has been employed. Metastasis has not been reported thus far so there is no role for adjuvant chemotherapy [4]. In unresectable de-novo or recurrent cases, radiation therapy has been used with favourable results [1, 4]. This report highlights the second known presentation of PHAT to the lower back, adding to the existing limited literature [2].
